# Oral bioavailability of cantharidin-loaded solid lipid nanoparticles

**DOI:** 10.1186/1749-8546-8-1

**Published:** 2013-01-08

**Authors:** Yun-Jie Dang, Chun-Yan Zhu

**Affiliations:** 1 Institute of Medicinal Plant Development, Chinese Academy of Medical Sciences and Peking Union Medical College, No. 151 Malianwa North Road, Haidian District, Beijing, 100094, P. R. China

## Abstract

**Background:**

The clinical application of cantharidin (CA) is limited by its insolubility, toxicity and short half-life in circulation. This study aims to achieve a steady and sustained blood concentration–time profile, using solid lipid nanoparticles (SLNs) as a drug carrier.

**Methods:**

CA-SLNs were prepared by a film dispersion–ultrasonication method. The physiochemical properties were studied by transmission electron microscopy. *In vitro* release and *in vivo* evaluation of CA-SLNs were studied by GC and GC-MS, while a comparison of the pharmacokinetic properties between CA-SLNs and free CA was performed in rats.

**Results:**

The mean size, drug content and encapsulation yield of CA-SLNs were 121 nm, 13.28 ± 0.12% and 93.83 ± 0.45%, respectively. The results show that CA-SLNs had a sustained release profile without a burst effect, a higher bioavailability than free CA after oral administration, and that the relative bioavailability of CA-SLNs to free CA was 250.8%.

**Conclusion:**

CA-SLNs could improve the solubility and oral bioavailability of CA.

## Background

**Figure 1:**
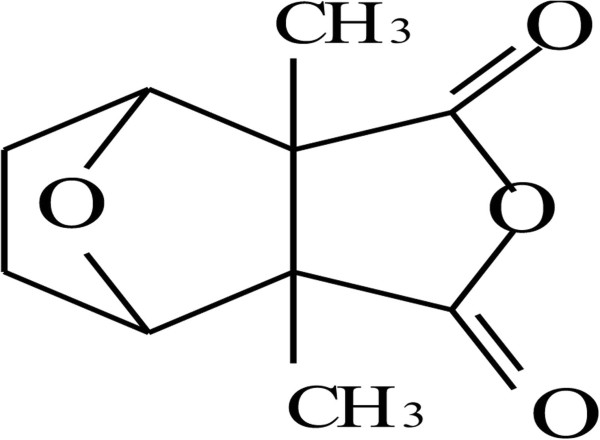
Structure of cantharidin.

Mylabris, the dry body of *Mylabris phalerata Pallas* or *Mylabris cichorii Linnaeus*, is recorded in the People’s Republic of China Pharmacopoeia as having an anticancer effect as well as the biological functions of eliminating toxic materials, eroding mycosis, eliminating blood stasis, and dispersing obstructions and lumps [1]. Recent research [2,3] indicates that cantharidin (CA; 2,3-dimethyl-7-oxabicyclo [2.2.l] heptane-2.3- dicarboxylic acid anhydride) is the principal active ingredient amongst the various compounds present in Mylabris (Figure 1). Pharmacological studies [4,5] show that CA can inhibit the growth of various implanted tumors in animal models by interfering with the metabolism of nucleic acids and proteins in cancer cells. It has inhibitory effects on primary hepatoma and other carcinomas, such as uterine cervix cancer, nasopharyngeal carcinoma, cutaneous cancer, and leukemia. The therapeutic efficacy of CA in the treatment of cancer and some refractory diseases had been demonstrated [6,7].

There are many kinds of Mylabris-based pharmaceutical preparations on the Chinese market, such as compound Mylabris injection (Aidi injection, State Permit No.Z52020236) and compound Mylabris capsules (State Permit No.Z19993294; State Permit No.Z200003270; State Permit No.Z20000427), all of which have proved to show good anticancer effects.

However, CA causes intense mucous membrane irritation [8]. It has been reported that mis-ingestion of CA causes the oral mucous membrane to ulcerate [9]. In clinical therapeutics, gastrointestinal indisposition is the main side effect of CA. The clinical use of cantharidin is limited by its short half-life and toxicity. In our previous work [10], CA was shown to be a poorly water-soluble drug with low oral bioavailability (26.7%) in beagle dogs. In addition, CA is a toxic anticancer drug [11], with a low lethal dose (30 mg) in humans and a median lethal dose of about 1 mg/kg [12]. Clinically, CA is administered in the dose range of 0.5 – 4 mg/d. It is necessary to find an efficient drug delivery system to reduce membrane irritation, control *in vivo* release and improve the bioavailability of CA.

Our research group has evaluated other preparation methods to reduce membrane irritation, such as solid dispersions [13] and inclusion complexes [14], both of which could improve bioavailability and reduce irritation to a certain extent but presented a rapid release and high maximum serum concentrations (C_max_).

In the pharmaceutical field, the merits of drug delivery systems in the nanosize range include increased solubility, reduced toxicity, and improved bioavailability [15]. Solid lipid nanoparticles (SLNs) are one example of a feasible nanosized drug carrier system. The particle matrix of a SLN is composed of solid lipid, which is very attractive for the controlled release of drugs [14] because drug mobility in a solid lipid should be considerably lower than in liquid oil, so that retarded drug release after oral administration can be achieved. Solid lipid nanoparticles combine the properties of polymer nanoparticles (a solid matrix for controlled release) and oil-in-water (o/w) type emulsions. A number of studies describing the production and physicochemical characterization of particles and associated drug incorporation and release have been published recently [16-21].

This study aims to improve the oral bioavailability of CA and control its release profile *in vivo* using an SLNs formulation, to study the physiochemical properties, and evaluate the *in vitro* release and *in vivo* behaviors of CA-SLNs.

## Methods

### Chemicals and reagents

A standard CA preparation (> 98% purity, 110783–200503) was purchased from the National Institute for Control of Pharmaceutical and Biological Products (Beijing, China). CA used for the preparation was purchased from Nanjing Zelang Medical Technological Development Co. Ltd in China (> 98% purity). Glyceryl monostearate was obtained from Hebei Gaobeidian Chunguang Chemical Reagent (China). Poloxamer 188 (F-68) was obtained from Sigma Chemicals (St Louis, MO, USA). Lecithin (Qrs95, Lot: A014989601) was purchased from Shanghai Taiwei Limited, Shanghai, China.

HPLC-grade methanol was purchased from Fisher Scientific Co. Ltd. (Dubuque, IA, USA). Absolute alcohol was of analytical reagent grade (Beijing Shiji, Beijing, China). All other reagents were of analytical grade (Beijing Chemicals, Beijing, China).

### Instrumental analysis

The exact drug content of the SLNs was determined using a pump L7100 and a Shimadzu SPD-10AVP UV-detector (Shimadzu, Japan). An SLC-10AVP (Shimadzu) equipped with a C-18 reversed-phase chromatographic column purchased from Dikma (China) (250 × 4.6 mm; 5 μm particle size) was used. The column was maintained at 30°C throughout the elution process, using a mobile phase comprising methanol and water 4:6 (v/v) at a flow rate of 1 mL/min. The detection wavelength was 228 nm. The presence of other materials did not interfere with the analysis of CA.

GC-MS was used to determine the concentration of CA in plasma. The GC/EI/MS system consisted of a Trace GC with a Thermo DSQ MSD (Thermo Finnigan, CA, USA). Samples were injected in splitless mode on a DB-5MS analytical column (30 m × 0.25 mm i.d. with 0.25 μm film thickness, J. & W. Scientific, USA). The carrier gas was helium with 99.999% purity at a flow rate of 1 mL/min. The injector temperature was set at 250°C. The column temperature started at 60 °C, was held for 1 min and then initially increased at 6°C/min to 220°C and then to 280°C at 20°C/min. The temperatures of both the source and MS Quad were at 250°C. The MS was operated in single ion monitoring (SIM) mode with electron impact ionization. CA and internal standard (IS; clofibrate) ion fragments monitored were m/z 128.

### Animals

Male Sprague–Dawley rats (SCXK (jing) 2007–0001; 200 – 250 g) were purchased from Vital River Laboratory Animal Technology Co. Ltd. (Beijing, China) for the animal experiments. Animals were kept in a normally controlled breeding room with standard laboratory food and water for one week prior to experiments. The rats were maintained according to internationally accepted principles of laboratory animal use and the study was approved by the Beijing Animal Care Committee. Twelve rats were randomly divided into two groups for the pharmacokinetic study. Another twelve rats were randomly divided into four groups for the irritation study.

### Preparation of the SLNs

Various samples of 2 mg cantharidin, 4 mg lecithin, 10 mg glyceryl monostearate and 4 mg cholesterol were dissolved in 10 mL alcohol, and the solution evaporated under reduced pressure to form a thin layer of uniform film at the bottom of the bottle. The residue of the aqueous phase, containing F-68 (0.5%) and Tween-80 (2%), were added to allow the film to expand and disperse for 30 min, and the mixture further dispersed ultrasonically for 60 min to obtain the CA-SLN suspension. On the basis of optimization for single factors, a central composite design was used for further optimization. The amounts of CA, F-68, lecithin, and glyceryl monostearate were used as the tested variables or independent variables, and encapsulation yield (EY), drug content (DC) as the evaluation indexes according to the following equations:

(1) $$\mathrm{EY}\left(\%\right)=\left(W_{T}-W_{F}/W_{T}\right)\times 100\%$$

(2) $$\mathrm{DC}\left(\%\right)=\left(W_{T}-W_{F}/W_{T}\right)\times 100\%$$

where W_T_ and W_F_ are the weights of total drug in SLNs and free drug in the ultra filtrate after centrifugation, respectively; and W_O_ is the weight of nanoparticles.

### Transmission electron microscopy

SLN dispersions (10 μL) were placed on a copper network layer on a carbon film. The sample was dried under room conditions before imaging the SLNs with a transmission electron microscope (JEM-1400 Transmission Electron Microscope, JEOL Ltd., Japan), operating at an acceleration voltage of 200 kV.

### Determination of mean diameter and surface charge

The mean particle size (z-average) of the SLNs was measured by photon correlation spectroscopy (Malvern Mastersizer 2000, U.K.) by a helium-neon laser with a wavelength of 633 nm. Photon correlations of spectroscopic measurements were carried out at a scattering angle of 90°. A 1:50 dilution of the formulations was made with double distilled water before measurement. The zeta potential of CA-SLNs was determined with a zeta potential instrument (32BIT, Brookhaven, MA, USA).

### Drug encapsulation efficiency and drug loading content determination

Free CA (not loaded within the CA-SLNs) was separated by an ultrafiltration centrifugation technique (HERAEUS Labofuge 400R, Thermo Scientific, U.S.A.). 2 mL of SLN solution loaded CA was added to the centrifugation technique and centrifuged at 12500 × *g* for 15 min at 4°C and the supernatant was then transferred to a clean tube and filtered with a 0.45 μm membrane. TrionX-100 (0.5 mL, 10%) was added into 2 mL of CA-SLNs colloidal solution and vortexed for 5 min to obtain the total CA. Free and total CA concentrations in the CA-SLNs were measured by HPLC.

The CA-SLNs colloidal solution was withdrawn by ultracentrifugation and freeze-dried to determine the drug loading content by accurately weighing the residue. The EY and DC were then calculated using equations (1) and (2), respectively.

### Mucous membrane irritation experiment

Four groups of three rats had free access to water but were fasted for 24 h before drug administration and for 4 h after drug administration. All samples including physiological saline (negative control group), salicylic acid, free CA and CA-SLN, were dispersed separately in 2 mL of physiological saline and then administered orally. The rats were killed *via* CO_2_ inhalation. The intact stomach was removed and cut into pathological sections.

### *In vitro* drug release

CA release from SLNs was performed in phosphate buffered saline (PBS; pH 6.8) by a Franz diffusion cell. A cellulose membrane was mounted between the donor and receptor compartments. The donor medium consisted of 2 mL CA-SLNs solution. The receptor medium consisted of 8 mL of 1.5% PBS in pH 6.8 buffer to maintain sink conditions during the experiments. The available diffusion area between cells was 2.84 cm^2^. The stirring rate and temperature were kept at 100 rpm and 37 ± 5°C, respectively. At appropriate intervals, 200 μL aliquots of the receptor medium were withdrawn and immediately replaced with an equal volume of fresh buffer. The amount of drug released was determined by HPLC. All operations were carried out in triplicate.

### *In vivo* administration studies

Two groups of six Sprague–Dawley rats were allowed free to access to water but were fasted for 24 h before drug administration and for 6 h after drug administration. CA and CA-SLNs were dispersed in 2 mL of physiological saline and then administered orally. The doses were 0.1 mg·kg^-1^ as CA. Blood samples were withdrawn from the jugular vein of rats at 0.5, 1, 2, 3, 4, 6, 8, 10, and 12 h after dosing. Aliquots of 0.2 mL of plasma samples from rats in a disposable Eppendorf tube, were combined with 200 μL HCl (6 M), 28 ng of IS (2.8 μg·mL^-1^) and 2 mL ethyl acetate. After vortexing for 90 s, the tube was centrifuged at 12500 × *g* for 15 min. The supernatant (0.8 mL) was transferred to a clean tube and evaporated to dryness under a stream of nitrogen gas at 30°C. The residue was dissolved in 50 μL ethyl acetate and then injected into the GC-MS system for analysis.

The AUC (area under the time-concentration curve) was calculated using the linear trapezoidal rule (Microsoft Office Excel 2007, U.S.A.) from zero to the last plasma concentration. The maximum plasma concentration, C_max_, and the time at which it occurred, T_max_, were obtained directly from the actual observed data.

## Results and discussion

Pharmacology studies [3-5] prove that CA can interfere with the metabolism of nucleic acids and proteins in cancer cells, significantly inhibiting the growth of various implanted tumors in animal models. Although CA has been shown to possess a strong anticancer effect, its poor oral absorption and the intense mucous membrane irritation it can cause may limit its clinical efficacy. In this study, we used SLNs, as an alternative drug carrier system to increase absorption, reduce toxicity and improve bioavailability.

### SLN properties

**Figure 2:**
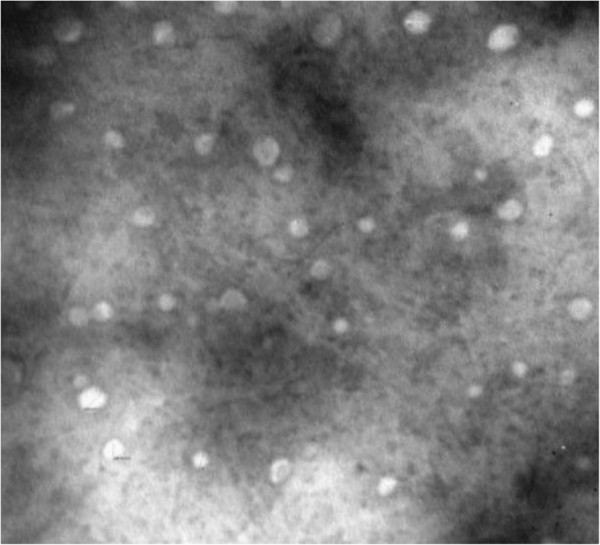
Electron microscope image of CA-SLNs.

The prepared CA-SLNs were round and essentially uniform (Figure 2), with a mean diameter of 121 nm. The zeta potential was −23.09 ± 0.53 mV and they were stable in solution. An EY of 93.83 ± 0.45% and a DC of 13.28 ± 0.12% (mean ± SD, *n* = 3) were obtained. The preparative technology was shown to be reproducible, because the values of EY and DC were both precisely expressed with a relative standard deviation of less than 2.5%.

**Figure 3:**
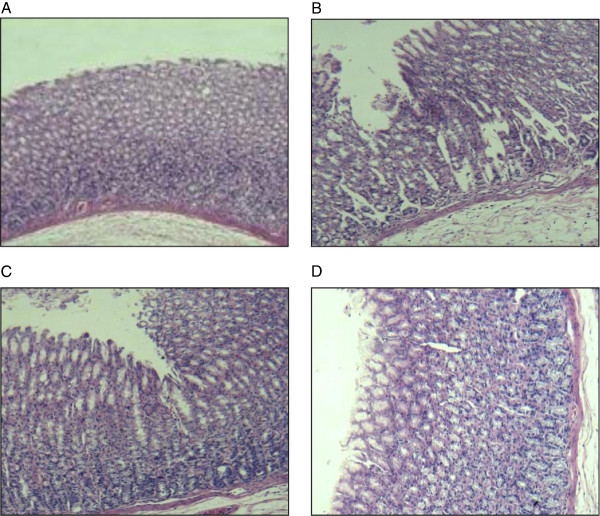
**The profiles of mucous membrane irritation experiments.** (**A**) negative control group (**B**) positive control group (**C**) cantharidin (**D**) CA-SLNs.

Figure 3 indicates that free CA caused gastrointestinal mucous membrane irritation (Figure 3C) but the irritation was relieved after encapsulation in SLNs (Figure 3D). This may be attributed to the drug entering the HP-β-CD cavity without coming into direct contact with the gastrointestinal mucous membrane.

### *In vitro* dissolution release

**Figure 4:**
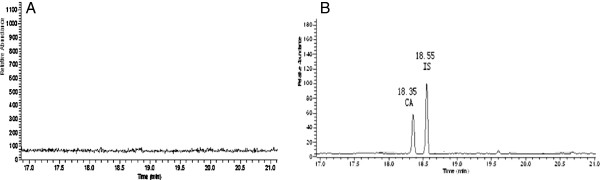
Release profiles of free CA and CA in CA-SLNs in PBS (pH 6.8) at 37°C (n = 3).

Figure 4 indicates that the amount of CA dissolved from the solid lipid nanoparticles was significantly higher than that of free CA at each time point. This phenomenon could be attributed to the nano-size of CA [22].

### *In vivo* administration studies

A sensitive GC-MS method was successfully applied to determine the plasma concentration of CA in rats after administration. The time-course concentration data was analyzed for bioavailability by WinNonLin (version 4.0. Pharsight Corperation, USA).

**Figure 5:**
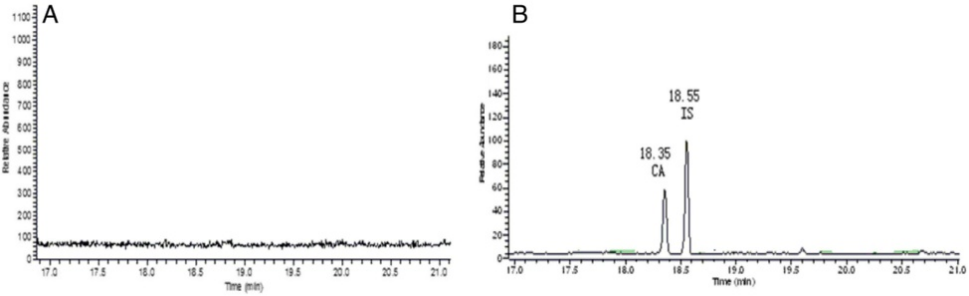
**Representative SIM (m/z = 128) chromatograms of CA and IS.** (**A**) Blank plasma; (**B**) Typical chromatograms of CA and IS in plasma.

**Figure 6:**
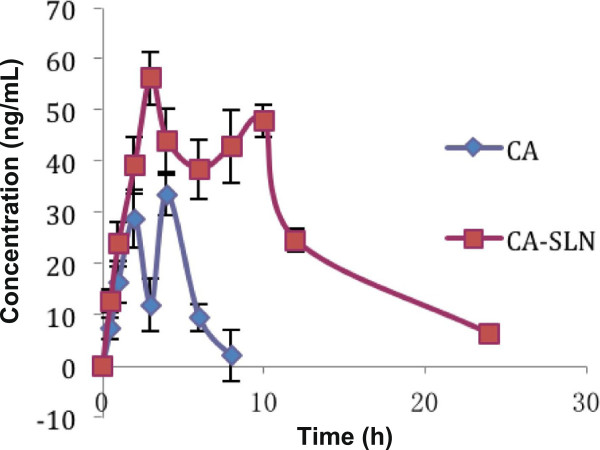
The CA plasma drug concentration–time profile for rats (0.1 mg/kg; n = 5).

Figure 5 shows typical single ion measurement (SIM) (m/z = 128) chromatograms for CA and clofibrate in plasma. As shown in Figure 6 and Table 1, after a single dose of CA (0.1 mg/kg), the C_max_ value from CA-SLNs was higher than that of CA, and a prolonged drug release was obtained.

**Table 1:** Pharmacokinetic parameters of CA in two preparations (n = 5)

	**C**_**max **_**(ng/mL)**	**T**_**max **_**(h)**	**AUC**_**0~t **_**(ng/mL)·h**	**Fr (%)**
CA	66.21 ± 29.17	2.33 ± 0.56	187.37 ± 76.09	100.0
CA-SLN	106.57 ± 17.56	2.67 ± 0.57	470.01 ± 146.94	250.8

Two plasma peaks were observed after the administration of CA-SLNs. The first peak was attributed to the presence of free drug and the burst release, while the second peak was attributed to the controlled release or potential gut uptake of SLNs. The occurrence of these two peaks was also reported by Yang *et al.*[23].

The bioavailability data show that the adsorption of CA-SLNs was 250.8%, which was higher that of free CA *in vivo*, corresponding with the release result determined *in vitro.* The different absorption rates between CA and CA-SLNs could be due to the nanoparticle size and the influence of the lipid carrier.

## Conclusion

CA-SLNs could improve the solubility and oral bioavailability of CA.

## Abbreviations

CA: Cantharidin; Cmax: the maximum concentration; AUC: the area under the time-concentration curve; GC-MS: Gas chromatography mass spectrometry; SIM: Single ion measure; IS: Internal standard; RSD: the relative standard deviation.

## Competing interest

The authors declare that they have no competing interests.

## Authors’ contributions

YJD designed the study, conducted the experiments and drafted the manuscript. CYZ conceived and participated in its design. All authors read and approved the manuscript.
